# Food safety knowledge, attitudes and practices of food handlers: A cross-sectional study in school kitchens in Espírito Santo, Brazil

**DOI:** 10.1186/s12889-021-10282-1

**Published:** 2021-02-12

**Authors:** Alyne Gomes da Vitória, Jhenifer de Souza Couto Oliveira, Louise Caroline de Almeida Pereira, Carolina Perim de Faria, Jackline Freitas Brilhante de São José

**Affiliations:** 1 Student of Nutrition and Health Postgraduate Program, Federal University of Espírito Santo, Avenida Marechal Campos, 1468, Vitória, 29040-090 Brazil; 2 Department of Integrated Education in Health, Federal University of Espírito Santo, Avenida Marechal Campos, 1468, Vitória, 29040-090 Brazil; 3 Nutrition and Health Postgraduate Program, Federal University of Espírito Santo, CEP 29043-910, Marechal Campos, 1468, Vitória, 29040-090 Brazil

**Keywords:** Food safety, Food handling, Food hygiene, School feeding, Food service, Collective feeding, Food quality

## Abstract

**Background:**

The adoption and evaluation of good practices in food handling in food service are essential to minimizing foodborne diseases. The present study aimed to evaluate food safety knowledge, attitudes, and practices of food handlers in schools in Vitória, Brazil.

**Methods:**

A cross-sectional study was carried out in the school food services of the municipal network of Vitória-ES. The sample of food handlers was obtained by convenience and comprised food handlers involved with preparation and other kitchen-related activities. The instrument consisted of a structured questionnaire with 36 six questions that included sociodemographic characteristics, knowledge, attitudes, and practices (KAP) related to good practices and food safety. The questionnaire was answered by 172 food handlers. Pearson correlation test, T-test, Tukey’s test and multiple linear regression analysis were conducted. Data entry and analysis were done using SPSS v.20 software.

**Results:**

Most of the participants were female (96.5%, *n* = 166), were 40 to 49 years old (44.8%, *n* = 78), attended high school (57.9%, *n* = 99), had up to 5 years of experience in the role (39.5%, *n* = 68). Some of them had participated at least 4 times in training (74.4%, *n* = 128) of which the most recent session had occurred within 3 months (52.0%, *n* = 44). The lowest score was obtained for knowledge (7.1 ± 1.22). All the models presented significant results for the F-test. This result show good model fit and results ranging from 1.5 to 2.5 on the Durbin Watson test of residual autocorrelation. The linear regression analysis allowed us to identify that the knowledge score increased with experience, but it was significant only for those who had spent up to 10 years in the role. The knowledge score was associated with experience and training time. Attitudes were significantly related to the schooling and training time. The increase in the classification of practices is shown only through a classification of attitudes.

**Conclusions:**

Although the food handlers’ knowledge level in general was considered as sufficient, it was inferior to their scores for attitudes and practices regarding certain food safety concepts. Food safety training is ongoing in these units and covers the main aspects that favour the transformation of knowledge into appropriate attitudes and practices.

**Supplementary Information:**

The online version contains supplementary material available at 10.1186/s12889-021-10282-1.

## Background

According to the World Health Organization (WHO), millions of people are affected annually by diseases associated with the consumption of contaminated food, particularly in developing countries. These illnesses mainly affect children and other vulnerable groups, such as pregnant women, the sick and the elderly [1].

In the Brazilian context, children’s vulnerability is linked to another concerning issue, according to data from the Ministry of Health, the fifth most frequent location of outbreaks of foodborne diseases (FDs) in nurseries and schools [2]. The adoption of correct food handling practices is recommended by the legislation in force and covers a series of determinations. Precautions in food handling are necessary and must be adopted by all food service facilities, including school kitchens, to minimize the risk of FD occurrence [3].

Considering these aspects, the evaluation of the factors involved in safe food production is of great importance. Good practices contribute to one principle of the National School Feeding Program (NSFP), which aims to meet the needs of students through the provision of healthy and safely handled food. It is one of the largest school food programs in the world and is the only such program with universal participation [4].

Quality control of school meals is imperative because dangers from different sources can cause contamination between the food preparation and distribution stages and culminate in the occurrence of FDs. FDs are a major consequence of the lack of sanitary control in food service environments [5, 6].

Although food safety in food services is a relevant issue and measures are taken to guarantee food quality [7–9], studies conducted in different Brazilian locations have reported that food handlers’ behavior has an important influence on contamination and can reduce the quality of the final products [7, 10–13]. Then, food handlers have different food safety knowledge levels, and sometimes, an adequate knowledge level does not translate into good hygienic practices when processing and handling food products [13–16]. Thus, training programs contribute to knowledge about food safety, although knowledge acquisition does not always result in positive changes in good handling practices [14–16]. Given food handlers’ role in improving hygiene and sanitation in School Feeding Service (SFS) and considering the vulnerability of the public served by NSFP, the present study aimed to verify the level of food safety knowledge, attitudes and practices (KAP) among food handlers in schools in Vitória, Brazil. We aimed to verify three hypotheses in this study: i) food handlers don’t have a satisfactory knowledge level; ii) food handlers don’t have a satisfactory attitudes and practices level; iii) sociodemographic variables are related with food handler’s knowledge, practices and attitudes.

## Methods

### Study design

A cross-sectional study was conducted to evaluate the KAP related to food safety through a specific questionnaire for food handlers. This work is part of a larger project entitled “Evaluation of the level of knowledge, attitudes and practices of food handlers in food services”, which was presented to and approved by the Municipal Secretary of Education (MSE) of Vitória-ES. Following this approval, invitation letters were e-mailed to school managers with the MSE’s authorization to commence the project. The managers were also contacted via telephone or in person for permission to visit the schools.

#### Study area, sample size and sampling

Data were collected at SFS from schools within the municipal network in Vitória, Espírito Santo, Brazil. There are 100 municipal schools in Vitória, Brazil and all were invited to participate in this study. The school units are distributed among nine administrative regions. The composition of the sample was determined by considering the total number of school units and the proportion of units in each administrative region. The participation of 50% of the schools in each region was required to demonstrate representativeness. Fifty-two eligible schools were sampled using simple cluster sampling; schools were stratified according to the regions of the municipality and randomly selected from each region. The municipal school units are distributed among nine administrative regions: Region 1 – Total = 8 (*n* = 4); Region 2 – Total = 15 (*n* = 8); Region 3 – Total 16 (n = 8); Region 4 – Total = 22 (*n* = 12); Region 5 – Total = 2 (n = 1); Region 6 – Total = 7 (n = 4); Region 7 – Total = 18 (*n* = 9); Region 8 – Total = 6 (*n* = 3); Region 9 – Total = 6 (n = 3). All administrative regions are located in the urban area and the region 5 and region 9 have the highest incomes in the city.

The sample of food handlers was obtained by convenience and comprised those carrying out food preparation and other kitchen-related activities in 52 municipal schools. All food handlers who were available at the time of collection in schools were invited to participate. Each school had 2 to 5 food handlers.

#### Instrument for data collection

The KAP questionnaire applied in this research was subjected to a reproducibility test given the limitations associated with the use of such instruments, such as imprecise answers and failure to understand the material. This process allows the reproducibility levels of a questionnaire to be determined, which leads to obtaining better quality data [17].

Test-retest reliability was determined with 29 food handlers from one food service unit and were not part of the research sample. The questionnaires were administered at the participants’ workplace, and the retest procedure took place 15 days after the first administration.

The instrument consisted of a structured questionnaire based on related studies [15, 18, 19]. The content related to KAP issues and the correct answers was determined considering the Brazilian resolution of good practices for food service [3], the Codex Alimentarius [20], and the five keys to safer foods established by the WHO [21] and adapted from Cunha et al. [15]. Additionally, six questions assessed the following sociodemographic characteristics of the handlers: age, sex, education, participation in food safety training and amount of experience as a food handler.

The KAP evaluation was organized into three blocks following Cunha, Stedefeldt & Rosso [15]. The block related to knowledge evaluation comprised 10 objective questions related to the daily practices of food preparation and addressing the concepts of personal hygiene, food hygiene, cross-contamination and the thawing of food. The three answer options were “yes”, “no” and “I do not know”.

The attitude assessment block included 10 questions related to the importance of hygiene procedures, food handlers’ responsibility for avoiding foodborne illnesses and the importance of ongoing training about food safety. In this block, attitude was considered a way of thinking that is reflected by a person’s behavior. The food handlers indicated their level of agreement on a three-point scale that reflected the following response options: “I agree,” “disagree,” and “I do not know.”

The last block of the questionnaire referred to the evaluation of self-reported practices and comprised 10 questions about daily practices that addressed the same themes as the knowledge block. A five-point rating scale (1 = never, 2 = rarely, 3 = sometimes, 4 = often and 5 = always) was used to evaluate each practice. For practices that are considered inadequate, the scale was scored following an inverse order.

For the knowledge questions, one point was assigned for each correct answer; each incorrect or “I do not know” answer received zero points. The range of possible scores for the knowledge block was 0 to 10 points. The possible score for the attitude questions ranged from 0 to 100 points. For the practice’s questions, the possible score was from 10 to 50. For the evaluation of each block based on the sum of the final scores for each block, an adequate grade was 70% or higher based on a study by Soares et al. [19]. Completing the questionnaires took approximately 15 min and was performed by the participants themselves in the presence of the researchers. In situations of doubt or reading difficulties, the researchers read the questions to avoid providing further explanations that would influence the answers.

#### Data analysis

Data were tabulated in Microsoft Office Excel spreadsheets and analyzed using IBM SPSS Statistics software, version 22 (IBM Corporation, Armonk, NY, USA).

### Questionnaire reproducibility test

After an exploratory analysis of the data, reproducibility was assessed using the intraclass correlation coefficient and interpreted according to the criteria proposed by Cicchetti [22] using the following scale: poor (< 0.40), reasonable (between 0.40 and 0.59), good (between 0.60 and 0.74), and excellent (between 0.75 and 1.00).

#### Analysis of the data collected from the questionnaires

The normality of the data was tested with the Kolmogorov-Smirnov test, and when nonnormal distribution was present, the data were log normalized before the parametric tests were performed. Descriptive statistics were found using the frequency, percentage, mean, and standard deviation for the scores and sociodemographic characteristics.

To evaluate the correlation between the scores obtained for KAP, the Pearson correlation test (r) was performed considering the strength of the correlations and respective probability of errors (*p* ≤ 5%). The strength of the correlations was classified as negligible (0.01 to 0.09), low (0.10 to 0.29), moderate (0.30 to 0.49), substantial (0.5 to 0.69) and strong (≥0.70), as suggested by Davis [23].

T-test and analysis of variance (ANOVA) were conducted, followed by Tukey’s test, to compare the means of the KAP score while considering sociodemographic variables. A multiple linear regression analysis was performed to identify the variables that impacted the KAP scores. The model for the multiple linear regression analysis was established to identify the impact of the explanatory variables (schooling, experience, participation in training, time since the previous training, knowledge and attitudes) on KAP scores. All analyses adopted a significance level of 5%.

#### Ethical aspects

The participants were informed about the study objectives and methodologies and signed the Free and Informed Consent Form if they agreed to participate in the study. The study was approved by the Ethics and Research Committee of the Federal University of Espírito Santo (UFES) in number 1.632.711.

## Results

### Evaluation of the knowledge, attitudes and practices of food handlers

#### Questionnaire reproducibility

The reproducibility and internal consistency analyses showed that the questionnaire applied in the present study falls within the range of accepted repeatability. The intraclass correlation coefficient was 0.64.

### Application of the questionnaire

#### Sociodemographic characteristics of the food handlers

The sociodemographic variables obtained from 172 food handlers via the questionnaire are shown in Table 1. The majority (96.5%, *n* = 166) of the participants were female, aged between 40 and 49 years (44.8%, *n* = 78). Regarding education, most of the participants (57.9%, *n* = 99) attended high school, and 40.7% (*n* = 70) attended only elementary school.

**Table 1:** Socio-demographic characteristics of food handlers in 52 schools in Vitória, Espírito Santo, Brazil

Variable		n	%
Gender	Female	166	96.5
	Male	6	3.5
Age (years)	≤ 39	51	29.6
	40 to 49	78	45.4
	> 50	43	25.0
Education^a^	Elementary school	70	40.7
	High school	99	57.6
	University education	3	1.7
Experience (years)	Up to 5	68	39.5
	6 to 10	53	30.8
	> 11	51	29.7
Participation in training since started in this job	Up to 3 times	44	25.6
	4 times or more	128	74.4
Time of the previous training attended	Last 3 months Last 6 months 1 year or more	89 69 14	51.8 40.1 8.1

^a^ In each category are included food handlers with complete or incomplete education

Most of the participants had up to 5 years of experience in the role (39.5%, *n* = 68) and had participated in at least 4 training sessions (74.4%, *n* = 128), the most recent of which had occurred within 3 months (52.0%, *n* = 44).

### KAP questionnaire performance

An evaluation of the results obtained through the KAP questionnaire found that the lowest scores were obtained on the knowledge assessment block (73.3%) (Table 2).

**Table 2:** Score obtained in the evaluation of the knowledge, attitudes, and practices of the food handlers

Dimension	Reached 70% of grade (%)	Mean ± SD	Range minimum and maximum
Knowledge	73.3	7.1 ± 1.22	3–10
Attitudes	97.7	9.4 ± 0.98	5–10
Practices	99.4	47.2 ± 3.80	22–50

*SD* Standard Deviation

Boards 1, 2 and 3 present the results for the KAP questionnaire responses and their respective evaluation blocks (see Additional file 2). The questions that yielded a high percentage of correct responses in the knowledge-related block (Board 1) addressed the risk of food contamination from food handlers through disease, nonuse of good food-handling practices, and food defrosting and risk of disease due to the consumption of expired foods.

Question 1 on this topic (Board 1) had the highest proportion of incorrect answers (91.8%). Most of the participants stated that hand washing with soap is sufficient to avoid food contamination, which raises the question of whether the low number of correct answers was related to lack of knowledge (because they considered the use of detergent to be a correct practice) or was due to misinterpretation of the question.

In question 4 (Board 1), food handlers had the low number of correct answers (39%) may have been a consequence of doubt about the effects of the water phase change on microbiological risks.

Regarding the risks of using foods the day after their expiration date, addressed in question 7 (Board 1), 90.7% (*n* = 156) of the food handlers answered this question correctly. However, on question 6, only 25% (*n* = 43) of the participants reported that foods unfit for consumption always have a bad smell and a spoiled taste.

In contrast to the results for the knowledge block, the participants demonstrated good performance on the questions about attitudes (Board 2), especially question 10, to which all participants responded correctly. Only question 5 received less than 90% correct answers. A high percentage of correct responses (> 90%) was also observed by other authors [14, 15].

Among the most frequent correct practices by food handlers (Board 3) was the use of cleansing solutions when washing vegetables and fruits (91.9%, *n* = 158), addressed in question 6.

The correlation between the scores obtained for KAP was considered low (Table 3). Knowledge scores were not related to self-reported practices scores.

**Table 3:** Pearson’s correlation (r) among the scores obtained in the evaluation of KAP of food handlers

	Knowledge	*p*	Attitudes	*p*	Practices	*p*
Knowledge	–	–	0.158^a^	0.038	0.128	0.094
Attitudes			–	–	0.192^a^	0.012
Practices					–	–

^a^The correlation is significant at the 0.05 level according to Pearson’s correlation

Table 4 presents the comparison of the mean scores obtained by the food handlers considering sociodemographic variables. The data indicate significant differences in knowledge scores according to the amount of experience in the role and the time since the most recent training. A significant difference in attitudes was observed according to schooling and the time since the most recent training. There was no significant difference in the scores obtained for practices.

**Table 4:** Relationship between the scores obtained for knowledge, attitudes and practices of food handlers

Characteristic (***n*** = 172)	Knowledge			Attitudes		Practices	
	n	Mean ± SD	***p***	Mean ± SD	***p***	Mean ± SD	***p***
**Gender**			0.670		0.670		0.226
Female	166	7.10 ± 1.22		9.42 ± 0.99		47.17 ± 3.83	
Male	6	7.83 ± 1.33		9.33 ± 0.82		49.17 ± 2.04	
**Age (years)**			0.598		0.088		0.433
18–39	51	7.04 ± 1.50		9.35 ± 0.63		47.78 ± 2.56	
40–49	77	7.17 ± 1.12		9.35 ± 1.01		47.06 ± 4.40	
> 50	43	7.16 ± 1.05		9.23 ± 1.231		46.86 ± 3.9	
**Education Level**			0.154		**0.030**^a^		0.937
Elementary School	69	6.9 ± 1.20		9.17 ± 1.19^a^		47.13 ± 3.37	
High School	99	7.26 ± 1.25		9.58 ± 0.80^b^		47.27 ± 4.14	
University Education	3	7.67 ± 0.58		9.67 ± 0.58^ab^		48.00 ± 2.65	
**Experience (years)**			**0.036**^a^		0.472		0.995
Until 5	68	6.88 ± 1.38^a^		9.51 ± 0.84		47.19 ± 3.50	
6 a 10	53	7.45 ± 1.25^b^		9.32 ± 1.22		47.32 ± 4.44	
Equal or more than 11 years	51	7.10 ± 0.88^ab^		9.37 ± 0.90		47.25 ± 3.56	
**Training participation**^a^			0.117		0.570		0.869
Until 3 times	44	6.93 ± 1.34		9.41 ± 0.88		47.16 ± 3.69	
4 times or more	128	7.19 ± 1.18		9.41 ± 1.02		47.27 ± 3.85	
**Time until last training**			**0.039**^a^		**0.001**^a^		0.318
3 months	89	7.25 ± 1.26^a^		9.64 ± 0.67^a^		47.67 ± 2.90	
6 months	68	7.10 ± 1.14^ab^		9.07 ± 1.25^b^		46.84 ± 4.64	
1 year or more	14	6.36 ± 1.28^b^		9.41 ± 0.97^ab^		47.27 ± 3.79	

*SD* standard deviation; *p:* value of significance. Means followed by the same letter do not differ from each other, by the Tukey test (*p* < 0.05)

^a^ since food handler started in this job

The model for the multiple linear regression analysis was established to identify the impact of the explanatory variables (schooling, experience, participation in training, time of the previous training, knowledge and attitudes) on KAP scores. For this analysis, only the variables that presented statistically significant results were included in the bivariate analysis. To identify the association between the variables, the KAP score considered the assumption of the effect of knowledge on the change in attitudes and practices as well as the influence of attitudes on practices.

All the models presented significant results on the F-test, indicating good model fit, and results ranging from 1.5 to 2.5 on the Durbin Watson test of residual autocorrelation.

The linear regression analysis (Table 5) allowed us to identify that the knowledge score increased according to greater experience, but this increase was significant only for those who had spent up to 10 years in the role.

**Table 5:** Linear regression analysis between scores obtained for knowledge, attitudes and practices and sociodemographic variables

	Knowlegde				Attitudes				Practices			
	β	***p***	IC 95%	β ajusted	β	***p***	IC 95%	β ajusted	β	***p***	IC 95%	β ajusted
**Education Level**												
Elementary School												
High School					0.40	**0.00**	0.10; 0.70	0.34				
University Education					0.49	0.39	- 0.63; 1.62	0.41				
**Experience (years)**												
Until 5												
6 a 10	0.57	**0.01**	0.13; 1.00	0.60								
Equal or more than 11 years	0.21	0.33	- 0.22; 0.65	0.24								
**Time until last training**												
3 months												
6 months	- 0.14	0.46	- 0.52; 0.24	- 0.17	- 0.56	**0.00**	- 0.86; − 0.26	- 0.53				
1 year or more	- 0.89	**0.01**	- 1.5; − 0.20	- 0.91	- 0.06	0.81	- 0.60; 0.47	- 0.04				
**Knowledge**									0.42	0.07	- 0.37; 0.89	0.32
**Attitudes**									0.86	**0.00**	0.29; 1.43	0.79

*SD* standard deviation; *p:* value of significance

## Discussion

About questionnaire reproducibility, intraclass correlation coefficient was a good index of reproducibility according to Cicchetti [22]. Bas et al. [18], Nee and Sani [24], Halim et al. [25] and Mohd et al. [26] also tested the reliability of the questionnaires with food handlers and found good indexes of between 0.70 and 0.78.

Majority of food handler were female, aged between 40 and 49 years and attended high school. These results are similar to those found in other studies [15, 19, 27, 28], which also observed a predominance of females in food services in schools. Food service sector is usually dominated by the female labor force. Although the inclusion of women in the labor market has been marked by several changes, reports still indicate that women predominantly work in fields associated with domestic employment, such as the preparation of food [29, 30].

Regarding education, most of the participants (57.9%) attended high school, and 40.4% attended only elementary school. These levels of schooling are characteristic of the profile of these professionals, as shown in other Brazilian studies [15, 19] and studies in other countries [27]. Brazilian legislation does not establish a specific schooling level for food handlers [3]; however, it requires that these professionals be subject to periodic training. Because this work does not require a high level of education and qualification, remuneration is low. This factor negatively affects the training and interventions performed in food services because it can influence the motivation of workers and consequently interfere with the adoption of appropriate attitudes and practices [31, 32]. There is a linear relationship between food handlers’ educational level and the implementation of good practices in food services. Consequently, access to food handler’s education levels is important when planning training strategies. According Akabanda et al. [33], training can improve the food safety knowledge of food handlers, but this does not guarantee a positive adjustment in food handling behavior and attitudes.

Most of the food handlers of this study had up to 5 years of experience in the role and participated in at least 4 training. Cunha et al. [15], Soares et al. [19] and Vo et al. [34] also reported a high number of food handlers who underwent training, indicating good compliance with Brazilian legislation [3] regarding periodic training for food handlers. Hygiene training and education can be understood as a planned learning event intended to improve their knowledge about work-related activities; it can also be viewed as a source of perpetual changes in practices and attitudes [32, 33]. It is a requirement in the food production environment and provides continuous improvement opportunities for food handlers. Instruction should be offered every 6–12 months and its efficacy must be evaluated. It is important to mentioned that food safety education need to be conducted with methods that encourage behavioral change and purchase practical abilities [35].

Results obtained through the KAP questionnaire indicated that the lowest scores were found on the knowledge block. A similar result was found in studies by Soares et al. [19] and Lee et al. [36], which verified that the participants’ level of knowledge was insufficient and moderate, respectively. It is important to highlight that within the food service environment, it is necessary to seek continuous improvement. These results point to the need for improvements in food handlers’ knowledge. The findings show that food handlers have adopted attitudes that helped produce safe food, but they provided incorrect answers to questions directly related to food quality control. According to Soares et al. [19], self-reported practices tend to be overstated by respondents, i.e., they responded what is probable rather than what they truly do within the food service environment. It is important to emphasize that the food handlers’ participation in this research and the fact that the questionnaire was self-applied may have influenced the large number of adequate answers.

Seven knowledge questions presented a high percentage of correct answers (Board 1). However, a question about hand hygiene has high percentage of incorrect answers. Highest proportion of food handlers stated that hand washing with soap is sufficient to avoid food contamination. According to Brazilian legislation, hand sanitation should be performed with an antiseptic and odorless liquid soap or an odorless liquid soap and an antiseptic product [3]. Incorrect knowledge and interpretation of food handling practices could lead to lower awareness of good handling procedures and false ideas about food safety [16]. It is important to mention that the question about hand washing may have been misunderstood by food handlers. The lack of hand hygiene is a critical aspect. Food handlers’ hands can be as vectors in the spread of foodborne diseases due to inadequate individual hygiene or cross contamination behavior [37–39].

Although the subject of hand hygiene is constantly addressed with food handlers, this does not guarantee that will perform the procedure correctly and then can be a source of contamination. This fact can be justified by the food handlers’ low perception of the risks associated with incorrect practices or by work overload that causes employees to prioritize other activities that are considered more relevant [15]. Adopting correct hand hygiene practices is essential because failures of personal hygiene can cause food handlers to become sources of pathogenic microorganisms and cross-contamination [18]. Appropriate hand washing practices by food handlers can significantly decrease the risk of diarrheal disease and other foodborne diseases [33].

Another question with incorrect answers was related to the quality of water. According to legislation, ice for use in food must be made from drinking water and maintained in hygienic and sanitary conditions to prevent contamination [3]. Although the use of ice was been observed in the visited SFS, it is imperative that the entire food safety concept is conveyed to food handlers. Water supply is a relevant aspect, since is one of the main causes of foodborne diseases outbreaks in Brazil.

Food handlers reported that contaminated food always have a bad smell and a spoiled taste. This finding represents a relevant problem because it indicates that the food handlers do not perceive the risks associated with using contaminated foods. This result similar to those of Soares et al. [19] in a study of 166 food handlers in public schools in Camaçari, Bahia, in which only 16.3% of the participants were aware that contaminated food does not necessarily show changes in color, odor or taste. A different result was obtained by Walker et al. [35], in which 57% of the participants stated that they would know if the food were contaminated via sensory verification.

About attitudes, food handlers presented a better result than knowledge block (Board 2). A high percentage of correct responses for attitudes (> 90%) was also observed by other authors [14, 15]. According Akabanda et al. [33], the food handlers’ attitudes can influence the occurrence of foodborne diseases. Thus, they need to follow the food safety plans. However, it is important to declare that the attitudes were self-reported. Thus, there is a possibility that the participants answered something that in their day-to-day lives they do not effectively accomplish.

Practices evaluation about washing food was considerably higher than that obtained by Soares et al. [19]. These authors found that 48.2% of the participants conducted incorrectly sanitization procedure because the great majority did not have a consistent supply of cleanser in the SFS. The attitudes of food handlers are known to be important in the application of knowledge and can have a significant impact on individuals’ behavior and practices [36]. The inadequate of knowledge level can culminate to poor hygienic practices by food handlers [33]. However, food handlers’ reported practices may not be essentially coherent with procedures performed during food handling. Inspiration and motivation during hygiene training and education could be a strategy to positively affect attitudes and practices and conduct to an appropriate behavior on kitchens. It is important to mentioned that food handlers may have an over-report of good performances contrasted to their usual practices when not asked or observed.

In this study, knowledge scores were not correlated to self-reported practices scores. This corroborating the results obtained in studies by Cunha et al. [15] and Park, Kwak & Chang [40]. However, contradictory results are described by Rahman et al. [41] and Vo et al. [34]. Rebouças et al. [42] did not observe a significant association between knowledge, attitudes and self-reported practices among food handlers, head chefs and managers in hotel restaurants in Salvador, Brazil. The low correlation between knowledge and attitude scores shows that the food handlers’ knowledge about food safety can influence their food handling attitudes. In other words, food handlers with low knowledge levels may have inappropriate attitudes.

Another point observed in this study was a significant difference in knowledge scores according to the amount of experience in the role and the time since the most recent training. A significant difference in attitudes was observed according to schooling and the time since the most recent training. There was no significant difference in the scores obtained for practices. Nee and Sani [24] observed that food handlers with less than one year of experience had lower scores for knowledge than those who had more than 6 years of experience. In addition, as the time since the previous training increased, the knowledge score decreased, becoming statistically significant when the training had been conducted more than 1 year previously. Cunha et al. [15] found a difference in knowledge scores between recently trained food handlers and those with a longer time interval since training (18, 24, 36 months), suggesting a possible recommendation of biannual training with a maximum interval of one year to maintain the food handlers’ working knowledge.

The results of this study also indicated that an increase in the level of schooling was associated with an increase attitude score. The results differ from those of other authors, who did not show a significant relationship between level of schooling and attitudes but did find a relationship between schooling and the knowledge and practices of food handlers [19, 35]. The reduction in the attitudes score was more significant among those who had undergone retraining in the previous 6 months. This result may have been influenced by the self-reported nature of these responses because the attitudes score was higher among those who had undergone training more recently (in the previous 3 months).

Given the results presented, suitable solutions are necessary. These results can contribute to future research as well as to the planning of training and guidance about food safety. Food handlers must receive information to apply it to their work routine.

The present study was subject to limitations, such as the impossibility of visiting all schools in the municipality and reliance on the answers of the participants. The food handlers may have answered some questions correctly, which may or may not truly reflect what they do on a daily basis. To get closer to the reality of food handlers’ practices, it would be necessary to observe their entire daily work routine. In addition, it is known that the presence of a researcher in the work environment may influence participants’ responses to a questionnaire.

## Conclusions

The results obtained in this study indicated that, although the level of knowledge of the participants in general was sufficient, it was inferior when compared to scores on the comprehension of attitudes and practices of the food handlers on certain concepts related to food safety. The association of the KAP score with the sociodemographic variables indicates the need for training programmes on good practices to consider these factors. In addition, the specifics (themes, difficulties, motivation) in the effectiveness of the program’s impact on knowledge acquisition must be taken into account but are mainly important in changing the attitudes, practices and understanding of the food handlers regarding their role in school food preparation.

In this context, the adoption of evaluative methods before and after training to identify the aspects to be improved and the relevance of the training programme for food handlers is suggested. An intervention strategy with the involvement of all social actors of National School Feeding Program is essential, given the importance of the program, the appropriate responsibilities within it and in view of the irregularities observed. Consequently, the results of improvements will be more effective. We recommended a training schedule for food handlers to guarantee their continued training in food safety. In addition, the professional nutritionists, who are responsible for monitoring this food service, should regularly supervise the routine of school kitchens. Intervention activities aimed at food safety must be constant and monitored, even during the work routine, so that, from the moment of identifying the failures, corrective actions occur immediately. Thus, in order to not only indicate the food handlers about the mistake, but also to guide him on why and the importance of correcting certain incorrect behavior.

## Supplementary Information

**Additional file 1.** Questionnaire: Evaluation of Knowledge, Attitudes and Practices of Food Handlers.

**Additional file 2. Board 1** Knowledge of food safety by food handlers from 52 schools in in Vitória, Espírito Santo, Brazil. **Board 2** Evaluation of food safety attitudes by food handlers from 52 schools in Vitória, Espírito Santo, Brazil. **Board 3** Evaluation of food safety practices by food handlers from 52 schools in Vitória, Espírito Santo, Brazil.

## Data Availability

The datasets used and analyzed during the current study are available from the corresponding author on reasonable request.

## Abbreviations

FD: Foodborne diseases
NSFP: National School Feeding Program
SFS: School Food Service
MSE: Municipal Secretary of Education
CECE: Centers for Early Childhood Education
MSEE: Municipal Schools of Elementary Education
KAP: Knowledge, attitudes and practices
