# Supplementary material for: Food safety knowledge, attitudes and practices of food handlers: A cross-sectional study in school kitchens in Espírito Santo, Brazil
Source: BMC Public Health. 2021 Feb 12;21:349. doi: 10.1186/s12889-021-10282-1 (PMC7881630; doi:10.1186/s12889-021-10282-1)
Supplement: Supplementary file 1 — Additional file 1. Questionnaire: Evaluation of Knowledge, Attitudes and Practices of Food Handlers. [file 12889_2021_10282_MOESM1_ESM.doc]

| **FEDERAL UNIVERSITY OF ESPÍRITO SANTO, BRAZIL**  **QUESTIONNAIRE**  **EVALUATION OF KNOWLEDGE, ATTITUDES AND PRACTICES OF FOOD HANDLERS**  *Da Vitória et al.* | | | |
| --- | --- | --- | --- |
| **SOCIODEMOGRAPHIC CHARACTERISTICS** | | | |
| **1)** **Gender:** ( ) Female ( ) Male | | | |
| **2)** **Age:** ( ) 18- 20 years ( ) 20-29 years ( ) 30-39 years ( ) 40-50 years ( ) over 50 years | | | |
| **3)** **Level of education:** | | | |
| ( ) Elementary school | | | |
| ( ) High school | | | |
| ( ) University education | | | |
| **4) Experience as food handler:**  ( ) Until to 5 years  ( ) 6- 10 years  ( ) Equal or more than 11 years | | | |
| **5) Participation in training** **since started in this job:**  ( ) Until to three time  ( ) Four times or more | | | |
| **6) Time of the previous training attended:**  ( ) Last 3 months  ( ) Last 6 months  ( ) 1 year or more | | | |
| **KNOWLEDGE ABOUT FOOD CONTAMINATION, DISEASES TRANSMITTED BY FOODS AND GOOD HANDLING PRACTICES.** | **Yes** | **No** | **I do not know** |
| Hand hygiene, which consists of washing hands gently with neutral detergent under running water and drying with a paper towel, can prevent food contamination. |  |  |  |
| A food handler with diseases, such as diarrhoea, influenza and sore throat, poses a risk of food contamination. |  |  |  |
| The use of adornments, such as earrings, rings, and watches, can cause food contamination. |  |  |  |
| Water can be a vehicle for disease transmission, but once it becomes ice, the risk of disease transmission is reduced. |  |  |  |
| Contact between raw and cooked foods, such as lettuce, which is used in the presentation of fried/cooked food, can contaminate the cooked food. |  |  |  |
| Foods unfit for consumption always have a bad smell and taste spoiled. |  |  |  |
| Using food one day after the expiration date, even when there is no change in smell and/or flavour, is a health risk. |  |  |  |
| Washing vegetables in running water and soaking them in water with vinegar is sufficient for making this food safe for consumption. |  |  |  |
| Defrosting can be performed in a basin with or without water in the sink or on a table or countertop that is not refrigerated (room temperature). |  |  |  |
| Consumption of undercooked food can lead to diseases that cause vomiting and diarrhoea. |  |  |  |
| **EVALUATION OF THE ATTITUDES OF FOOD HANDLERS.** | **Agree** | **Disagree** | **I do not know** |
| Always wash hands thoroughly before handling food. |  |  |  |
| Raw foods should be stored separately from cooked foods. |  |  |  |
| Thawed foods can be refrozen. |  |  |  |
| Wearing necklaces, earrings and rings makes food contamination possible. |  |  |  |
| Foods with past expiration dates should not be consumed even when there are no changes in their smell and taste. |  |  |  |
| Food handlers with injuries, bruises, or hand injuries should not touch or handle food. |  |  |  |
| It is important that I learn more about the safe handling of food to avoid contamination and diseases a part of my professional responsibilities. |  |  |  |
| Check the expiration date of the products and check that the packaging is in good condition. |  |  |  |
| The best way to defrost meats is in a bowl with water. |  |  |  |
| Proper hygiene of utensils and equipment that come into contact with food is necessary to reduce the risk of contamination. |  |  |  |

| **EVALUATION OF SELF-RELATED PRACTICES OF FOOD HANDLERS** | **Never** | **Rarely** | **Sometimes** | **Often** | **Ever** |
| --- | --- | --- | --- | --- | --- |
| Do you clean your hands properly before handling food? |  |  |  |  |  |
| Do you keep your nails short and unpolished and remove all adornments (earrings, rings, wedding rings, watches and bracelets) before handling food? |  |  |  |  |  |
| Do you handle food when you have diarrhoea or another illness or unprotected hands? |  |  |  |  |  |
| Do you thaw foods outside of refrigerated areas (room temperature)? |  |  |  |  |  |
| Do you check the shelf life of the food at the time of receipt? |  |  |  |  |  |
| Do you use cleansing products when washing vegetables and fruits? |  |  |  |  |  |
| Do you use foods with past expiration dates when the food is unaltered in its smell and taste? |  |  |  |  |  |
| Do you use the same cutting board and knife to prepare raw foods and cooked foods? |  |  |  |  |  |
| Do you check if the food is well cooked before it is served? |  |  |  |  |  |
| When you store food in the refrigerator, do you put it in the refrigerators covered or in covered containers? |  |  |  |  |  |
