# Supplementary material for: Food safety knowledge, attitudes and practices of food handlers: A cross-sectional study in school kitchens in Espírito Santo, Brazil
Source: BMC Public Health. 2021 Feb 12;21:349. doi: 10.1186/s12889-021-10282-1 (PMC7881630; doi:10.1186/s12889-021-10282-1)
Supplement: Supplementary file 2 — Additional file 2. Board 1 Knowledge of food safety by food handlers from 52 schools in in Vitória, Espírito Santo, Brazil. Board 2 Evaluation of food safety attitudes by food handlers from 52 schools in Vitória, Espírito Santo, Brazil. Board 3 Evaluation of food safety practices by food handlers from 52 schools in Vitória, Espírito Santo, Brazil. [file 12889_2021_10282_MOESM2_ESM.zip › Additional file 2/Board 1-1bmc okR3.docx]

**Board 1** Knowledge of food safety by food handlers from 52 schools in in Vitória, Espírito Santo, Brazil.

| **Questions** | | **Answers % (n)** | |
| --- | --- | --- | --- |
|  |  | **Correct** | **Incorrect** |
| **1** | Hand hygiene, which consists of washing hands gently with neutral detergent under running water and drying with a paper towel, can prevent food contamination. | 8.2  (14) | 91.8  (157) |
| **2** | A food handler with diseases, such as diarrhoea, influenza and sore throat, poses a risk of food contamination. | 97.1 (167) | 2.9  (5) |
| **3** | The use of adornments, such as earrings, rings, and watches, can cause food contamination. | 97.7 (168) | 2.3  (4) |
| **4** | Water can be a vehicle for disease transmission, but once it becomes ice, the risk of disease transmission is reduced. | 39.0 (67.0) | 61.0  (105) |
| **5** | Contact between raw and cooked foods, such as lettuce, which is used in the presentation of fried/cooked food, can contaminate the cooked food. | 77.9 (134) | 22.1  (38) |
| **6** | Foods unfit for consumption always have a bad smell and taste spoiled. | 25.0  (43) | 75.0  (129) |
| **7** | Using food one day after the expiration date, even when there is no change in smell and/or flavour, is a health risk. | 90.7 (156) | 9.3  (16) |
| **8** | Washing vegetables in running water and soaking them in water with vinegar is sufficient for making this food safe for consumption. | 84.3 (145) | 15.2  (26) |
| **9** | Defrosting can be performed in a basin with or without water in the sink or on a table or countertop that is not refrigerated (room temperature). | 95.3 (164) | 4.7  (8) |
| **10** | Consumption of undercooked food can lead to diseases that cause vomiting and diarrhea. | 97.1 (167) | 2.9  (5) |
