# Supplementary material for: Food safety knowledge, attitudes and practices of food handlers: A cross-sectional study in school kitchens in Espírito Santo, Brazil
Source: BMC Public Health. 2021 Feb 12;21:349. doi: 10.1186/s12889-021-10282-1 (PMC7881630; doi:10.1186/s12889-021-10282-1)
Supplement: Supplementary file 2 — Additional file 2. Board 1 Knowledge of food safety by food handlers from 52 schools in in Vitória, Espírito Santo, Brazil. Board 2 Evaluation of food safety attitudes by food handlers from 52 schools in Vitória, Espírito Santo, Brazil. Board 3 Evaluation of food safety practices by food handlers from 52 schools in Vitória, Espírito Santo, Brazil. [file 12889_2021_10282_MOESM2_ESM.zip › Additional file 2/Board 2bmc okR3.docx]

**Board 2** Evaluation of food safety attitudes by food handlers from 52 schools in Vitória, Espírito Santo, Brazil.

| **Questions** | | **Answers % (n)** | |
| --- | --- | --- | --- |
|  |  | **Correct** | **Incorrect** |
| **1** | Always wash hands thoroughly before handling food. | 99.4 (171) | 0.6 (1) |
| **2** | Raw foods should be stored separately from cooked foods. | 90.1 (155) | 9.9 (17) |
| **3** | Thawed foods can be refrozen. | 95.9 (165) | 4.1 (7) |
| **4** | Wearing necklaces, earrings and rings makes food contamination possible. | 91.9 (158) | 7.6 (13) |
| **5** | Foods with past expiration dates should not be consumed even when there are no changes in their smell and taste. | 85.5 (147) | 14.5 (25) |
| **6** | Food handlers with injuries, bruises, or hand injuries should not touch or handle food. | 90.7 (156) | 9.3 (16) |
| **7** | It is important that I learn more about the safe handling of food to avoid contamination and diseases as a part of my professional responsibilities. | 96.5 (166) | 3.5 (6) |
| **8** | Check the expiration date of the products and check that the packaging is in good condition. | 98.3 (169) | 1.7 (3) |
| **9** | The best way to defrost meats is in a bowl with water. | 93.0 (160) | 7.0 (12) |
| **10** | Proper hygiene of utensils and equipment that come into contact with food is necessary to reduce the risk of contamination. | 100.0 (172) | - |
