# Supplementary material for: Food safety knowledge, attitudes and practices of food handlers: A cross-sectional study in school kitchens in Espírito Santo, Brazil
Source: BMC Public Health. 2021 Feb 12;21:349. doi: 10.1186/s12889-021-10282-1 (PMC7881630; doi:10.1186/s12889-021-10282-1)
Supplement: Supplementary file 2 — Additional file 2. Board 1 Knowledge of food safety by food handlers from 52 schools in in Vitória, Espírito Santo, Brazil. Board 2 Evaluation of food safety attitudes by food handlers from 52 schools in Vitória, Espírito Santo, Brazil. Board 3 Evaluation of food safety practices by food handlers from 52 schools in Vitória, Espírito Santo, Brazil. [file 12889_2021_10282_MOESM2_ESM.zip › Additional file 2/Board 3bmc okR3.docx]

**Board 3** Evaluation of food safety practices by food handlers from 52 schools in Vitória, Espírito Santo, Brazil.

| **Questions** | | **Answers % (n)** | | | | |
| --- | --- | --- | --- | --- | --- | --- |
|  |  | **Never** | **Rarely** | **Sometimes** | **Often** | **Ever** |
| **1** | Do you clean your hands properly before handling food? | 1.2  (2) | - | 1.2  (2) | 24.4  (42) | 72.7  (125) |
| **2** | Do you keep your nails short and unpolished and remove all adornments (earrings, rings, wedding rings, watches and bracelets) before handling food? | 1.7  (3) | - | 1.2  (2) | 12.8  (22) | 84.3  (145) |
| **3** | Do you handle food when you have diarrhoea or another illness or unprotected hands? | 83.1 (143) | 4.1  (7) | 4.7  (8) | 2.9  (5) | 4.1  (7) |
| **4** | Do you thaw foods outside of refrigerated areas (room temperature)? | 87.8 (151) | 2.3  (4) | 1.7  (3) | 0.6  (1) | 7.6  (13) |
| **5** | Do you check the shelf life of the food at the time of receipt? | 0.6  (1) | - | - | 11.6  (20) | 87.8  (151) |
| **6** | Do you use cleansing products when washing vegetables and fruits? | 1.2  (2) | - | - | 7.0  (12) | 91.9  (158) |
| **7** | Do you use foods with past expiration dates when the food is unaltered in its smell and taste? | 93.6 (161) | 1.7  (3) | 1.2  (2) | - | 0.6  (1) |
| **8** | Do you use the same cutting board and knife to prepare raw foods and cooked foods? | 79.1 (136) | 2.9  (5) | 7.0  (12) | 2.3  (4) | 8.1  (14) |
| **9** | Do you check if the food is well cooked before it is served? | 2.3  (4) | - | - | 8.1  (14) | 89.0  (153) |
| **10** | When you store food in the refrigerator, do you put it in the refrigerators covered or in covered containers? | 1.2  (2) | - | 0.6  (1) | 7.0  (12) | 91.3  (157) |
